# Stellate cells are mesenchymal stem cells

**DOI:** 10.1186/2047-783X-19-S1-S6

**Published:** 2014-06-19

**Authors:** Claus Kordes, Iris Sawitza, Silke Götze, Diran Herebian, Dieter Häussinger

**Affiliations:** 1 Clinic of Gastroenterology, Hepatology and Infectious Diseases, Heinrich Heine University, 40225 Düsseldorf, Germany; 2 Department of General Pediatrics, Neonatology and Pediatric Cardiology, Heinrich Heine University, 40225 Düsseldorf, Germany

## Background

Vitamin A-storing hepatic stellate cells (HSC) are mainly known for their contribution to fibrogenesis in chronic liver disease, but their identity and function in normal liver remained unclear. Since HSC possess stem/progenitor cell characteristics [1] their contribution to stem cell-associated functions in the liver was investigated. Stem cell functions in the liver were reported during fetal hematopoiesis and tissue repair after severe injury when the proliferation of hepatocytes is impaired. By using several *in vitro* and *in vivo* approaches evidence was provided that stellate cells represent mesenchymal stem cells (MSC), which can support hematopoietic stem/progenitor cells in the fetal liver and contribute to the regeneration of injured liver as multipotent adult stem cells.

## Materials and methods

Hepatic and pancreatic stellate cells (HSC, PSC) were isolated from rats by enzymatic digestion of the tissue followed by density gradient centrifugation and characterized by vitamin A fluorescence, polymerase chain reaction (PCR) as well as immunofluorescence staining using molecular markers of stellate cells and bone marrow MSC. Rat HSC were co-cultured with hematopoietic stem cells, which were enriched from murine bone marrow by magnetic cell sorting using antibodies against SCA1 (stem cell antigen 1). Hematopoietic stem/progenitor cell markers were monitored by mouse-specific quantitative PCR during co-culture. Stellate cells from enhanced green fluorescent protein-expressing (eGFP^+^) rats were transplanted into lethally irradiated wild type rats to investigate their contribution to blood formation *in vivo*. Isolated stellate cells were treated with adipocyte or osteocyte differentiation media to evaluate a MSC-related differentiation potential. In order to investigate their contribution to liver regeneration, stellate cells from the pancreas of eGFP^+^ male rats were expanded in culture for 7 days and finally transplanted into female wild type rats that underwent partial hepatectomy (70% PHX) in the presence of 2-acetylaminofluorene (2AAF). Engraftment of eGFP^+^ stellate cells was analyzed during liver regeneration by qPCR of eGFP mRNA and sex-determining region Y gene (SRY DNA) as well as immunohistochemistry of eGFP and combined immunofluorescence of hepatocyte or bile duct markers.

## Results

Blood formation support is a typical function of bone marrow MSC. Desmin-expressing HSC are closely associated with GATA binding protein 1^+^ (GATA1^+^) cell clusters, which represent hematopoietic sites in the fetal liver, indicating that HSC may support blood formation. In order to investigate this, stellate cells from rat liver were co-cultured with SCA1^+^ hematopoietic stem cells from the bone marrow of mice. Murine hematopoietic stem cells formed colonies on rat stellate cells as feeder layers within 10 days of co-culture and maintained the expression of SCA1 as investigated by mouse-specific qPCR. Similar to rat bone marrow MSC, HSC were able to induce GATA1 expression in murine hematopoietic stem/progenitor cells, which indicated development of erythroid cells. In contrast to this, hematopoietic stem cells were poorly maintained on plastic without feeder layer. Among major liver cell types such as hepatocytes, sinusoidal endothelial cells and Kupffer cells (liver macrophages), only HSC significantly supported the maintenance of murine SCA1^+^ hematopoietic stem cells in co-culture. The supportive effect of HSC can be explained in part by the expression of cytokines and growth factors such as colony stimulating factors, interleukins and erythropoietin, which are known to influence hematopoiesis. Although HSC can support blood formation by hematopoietic stem/progenitor cells, they failed to reconstitute hematopoiesis after transplantation of eGFP^+^ stellate cells into lethally irradiated rats as known for bone marrow MSC. This finding indicates that the function of stellate cells during fetal hematopoiesis in the liver is restricted to supportive effects on blood formation. The classification of HSC as liver-resident MSC is further endorsed by an MSC-related expression profile (e.g. CD146, nestin, neuron-glia antigen 2 etc.) and the potential of HSC to differentiate into adipocytes and osteocytes *in vitro*[2]. Especially the differentiation into adipocytes and osteocytes is commonly used to identify MSC. Similar MSC-associated characteristics were found in PSC, suggesting that stellate cells may in general be viewed as MSC. Another important feature of stem cells is their contribution to tissue repair through differentiation into various effector cells. Growth factor (hepatocyte growth factor and fibroblast growth factor 4) treatment induced hepatic differentiation of isolated stellate cells from liver and pancreas, which acquired a typical expression pattern of liver parenchymal cells (e.g. albumin, hepatocyte nuclear factor 4α, cytochrome P450 7A1) [1,3] and released bile acids into the culture medium. This suggests that stellate cells may transiently become liver progenitor cells, which were termed oval cells in rodents, during differentiation into hepatocytes. Studies are underway to clarify the existence of an intermediate state of stellate cells and liver progenitor cells during hepatic differentiation *in vitro* and *in vivo*. A similar differentiation potential can be found in clones of stellate cells, which derived from single PSC expanded in culture. These clones maintain the expression of stellate cells while preserving their differentiation potential, which indicates self-renewal potential as a typical feature of stem cells [3]. Transplantation studies with eGFP^+^ PSC revealed that stellate cells are able to reconstitute the injured liver in host animals through the formation of various liver cell types such as hepatocytes and cholangiocytes. Preliminary results indicated a similar contribution of transplanted eGFP^+^ HSC to liver regeneration. In contrast to stellate cells, fibroblasts obtained from the abdominal muscle of eGFP^+^ rats by outgrowing failed to reconstitute the injured host liver [3].

## Conclusions

The findings reported herein justify a classification of stellate cells as liver- and pancreas-resident MSC. The classification of stellate cells as MSC is in good agreement with former observations that focused on their contribution on fibrogenesis, since a fibrogenic potential was also suggested for bone marrow MSC. In addition, the support of hematopoiesis in the fetal liver represents an important non-disease-related function of HSC. The provision of a suitable environment for maintaining the quiescent state of stellate cells as hepatic MSC [4] and supporting hematopoietic stem cells in liver sinusoids [2] indicate that the space of Disse, the intercellular space between sinusoidal endothelial cells and hepatocytes, represents another hepatic stem cell niche in addition to the canals of Hering [4,5]. However, the function of stellate cells is not restricted to supportive effects, since they can contribute to liver regeneration through differentiation into epithelial cell lineages.
